# The Role of Transthoracic Echocardiography in the Evaluation of Patients With Ischemic Stroke

**DOI:** 10.3389/fcvm.2021.710334

**Published:** 2021-08-27

**Authors:** Robson Sarmento Teodoro, Gisele Sampaio Silva, Gabriel Pinheiro Modolo, Stella De Angelis Trivellato, Juli Thomaz de Souza, Gustavo José Luvizutto, Hélio Rubens de Carvalho Nunes, Luis Cuadrado Martin, Rodrigo Bazan, Silméia Garcia Zanati Bazan

**Affiliations:** ^1^Department of Internal Medicine, Botucatu Medical School - Universidade Estadual Paulista “Julio de Mesquita Filho” (UNESP), São Paulo State University, Botucatu, Brazil; ^2^Department of Vascular Neurology, Universidade Federal de São Paulo (UNIFESP), São Paulo, Brazil; ^3^Department of Neurology, Botucatu Medical School - Universidade Estadual Paulista “Julio de Mesquita Filho” (UNESP), São Paulo State University, Botucatu, Brazil; ^4^Department of Applied Physiotherapy, Triangulo Mineiro Federal University, Uberaba, Brazil; ^5^Department of Biostatistics, Botucatu Medical School - Universidade Estadual Paulista “Julio de Mesquita Filho” (UNESP), São Paulo State University, Botucatu, Brazil

**Keywords:** stroke, ischemic stroke, echocardiography, cardioembolism, stroke etiology

## Abstract

**Background:** Ischemic stroke can be classified into five etiological types, according to the Trial of Org 10172 in Acute Stroke Treatment (TOAST) classification, and its adequate investigation and characterization can aid in its clinical management and in preventing new events. Transthoracic echocardiography (TTE) plays a key role in investigating its etiology; approximately one-third of the patients remain without an adequate definition of the etiology or are classified as the undetermined TOAST type.

**Objectives:** To evaluate if the percentage of patients with indeterminate etiology according to the TOAST classification decreased after transthoracic echocardiography, to determine whether or not the prognosis after ischemic stroke is worse among patients classified as the undetermined TOAST type, and to verify the predictive capacity of echocardiography on the prognosis after ischemic stroke.

**Methods:** In this retrospective cohort study, clinical, neurological, and echocardiographic examinations were conducted when the patient was hospitalized for stroke. In-hospital mortality and functional capacity were evaluated at hospital discharge and 90 days thereafter. Multiple linear regression and multiple logistic regression models were adjusted for confounding factors. The level of significance was 5%.

**Results:** A total of 1,100 patients (men = 606; 55.09%), with a mean age of 68.1 ± 13.3 years, were included in this study. Using TTE, 977 patients (88.82%) were evaluated and 448 patients (40.7%) were classified as the undetermined TOAST type. The patients who underwent TTE were 3.1 times less likely to classified as the undetermined TOAST type (OR = 0.32; *p* < 0.001). Echocardiography during hospitalization was a protective factor against poor prognosis, and reduced the odds of in-hospital death by 11.1 times (OR: 0.090; *p* < 0.001). However, the presence of the undetermined TOAST classification elevated the chance of mortality during hospitalization by 2.0 times (OR: 2.00; *p* = 0.013).

**Conclusions:** Echocardiography during hospitalization for ischemic stroke reduces the chances of an undetermined TOAST classification and the risk of in-hospital mortality. However, being classified as the undetermined TOAST type increases the chance of mortality during hospitalization, suggesting that evaluating patients using echocardiography during hospitalization for acute ischemic stroke is important.

## Introduction

A stroke is characterized by an acute neurological deficit attributed to a focal lesion of vascular origin in the central nervous system (CNS), which may be secondary to an ischemic infarction, or a parenchymal or subarachnoid hemorrhage (1). A CNS infarction is defined as the death of the brain, retinal, or spinal cord cells due to ischemia, as confirmed by pathological evidence on imaging examination. A CNS infarction may also be defined by other evidences of injury to the vascular territory or by the persistence of symptoms for more than 24 h after excluding other causes (1).

It is estimated that during their lifetime, one in six men and one in five women present with stroke (2), which is the second leading cause of death and is responsible for approximately one in eight deaths worldwide (3). In Brazil, stroke is the second leading cause of death and the leading cause of disability (4, 5).

Stroke can be classified according to the pathology, etiology, and clinical presentation (6). According to the pathological classification, a stroke may be hemorrhagic or ischemic, with the latter corresponding to 80% of the total stroke cases. Etiologically, ischemic stroke can be categorized into five types, according to the Trial of Org 10172 in Acute Stroke Treatment (TOAST) classification: (1) large-artery atherosclerosis, (2) cardioembolism, (3) small-vessel occlusion, (4) stroke of other determined etiology, and (5) stroke of undetermined etiology (7). The proportion of patients in each group differed among the studied populations. The definition of the etiological mechanism is important for evaluating severity, progression, and prognosis. The cardioembolic type is responsible for 14–30% of ischemic conditions and has higher mortality, greater severity, and worse functional outcome compared to the other etiologies (8, 9); the affected patients are predisposed to early recurrence. Atrial fibrillation (AF) is the main finding associated with this type of stroke.

Rücker et al. studied 3,346 ischemic stroke patients to determine the long-term survival and recurrence after ischemic stroke according to the etiological subtype (the TOAST classification) in a population-based stroke registry in Germany. Their study showed that the 5 year survival rate was higher in patients with stroke, due to the occlusion of the small arteries, and lower in patients with cardioembolic stroke. Furthermore, the 5 year recurrence rates were lower in women with stroke, due to small artery occlusion, and in men with large artery atherosclerosis. The highest recurrence rates, in both women and men, were seen in indeterminate stroke (10). Existing literature still reports a certain degree of conflict in the clinical prognosis, mortality, and recurrence rate in the undetermined TOAST type, and this can be attributed to the heterogeneity of this etiological subtype, which comprises different pathophysiological mechanisms.

The cardiovascular risk profile and echocardiographic findings in patients with AF detected after a stroke are comparable to those of patients previously diagnosed with AF, but differ from those of patients without AF. Preexisting heart disease is the major cause of AF and is first diagnosed after a stroke (11).

Some disorders are considered to be high-risk sources for the cardioembolic type, such as mitral stenosis, heart valve prosthesis, myocardial infarction in the previous 4 weeks, mural thrombus in the left cavities, left ventricular aneurysm, any documented history of permanent or transient fibrillation or atrial flutter with or without spontaneous contrast echocardiogram or left atrial thrombus, sinus node disease, dilated cardiomyopathy, ejection fraction <35%, endocarditis, intracardiac mass, patent foramen ovale with *in situ* thrombosis, and patent foramen ovale associated with pulmonary thromboembolism or peripheral venous thrombosis prior to the ischemic stroke (12).

Furthermore, with regard to structural heart diseases, four studies considered left ventricular dysfunction defined as recent heart failure, a 25% reduction in left ventricular ejection fraction, and an ejection fraction inferior to 50% as independent risk factors for stroke, despite a population overlap in three of the four studies. Two of the studies also considered ventricular hypertrophy and a left ventricular mass >110 g/m^2^ in women and 134 g/m^2^ in men as independent risk factors for stroke (13).

Left atrial enlargement is an independent factor for stroke and is associated with a 20% chance of thromboembolism per year in the presence of a left atrium >2.5 cm/m^2^ with moderate to severe left ventricular contractility changes (14).

Left ventricular dysfunction and left atrial size were the strongest independent predictors of late thromboembolism. Patients without these two predictors on echocardiography, or without the three identified clinical predictors of thromboembolism (history of hypertension, recent heart failure, and previous thromboembolism) had a low risk of thromboembolism (1% per year). However, patients with no thromboembolism predictors but with one or both echocardiographic predictors had a 6% risk of stroke per year, showing that in addition to clinical assessment, echocardiography can stratify patients with AF and guide their therapy (15).

Despite all investigations, about a third of ischemic stroke patients cannot be categorized etiologically and are classified as the undetermined TOAST type, which can comprise potential cardiac sources of embolism, atherothrombotic causes, and cerebral embolism from indeterminate sources (16). The American Heart Association and American Stroke Association (AHA/ASA) guidelines recommend echocardiography for evaluating a patient with ischemic stroke only in selected cases (class IIa/class IIb). A recent study published by Harris et al. aimed to investigate the utility of transthoracic echocardiography (TTE) as a part of an acute ischemic stroke workup and revealed that the overall yield of TTE in acute ischemic stroke was low (17). Conversely, TTE has been performed as part of the assessment of stroke patients in recent years. More recently, point-of-care ultrasound (POCUS) has increased its field of application and TTE has been used as a screening method in the stroke unit (18). Robust registries, such as The Cornell Acute Stroke Academic Registry (CAESAR), routinely perform echocardiography as a strategy for evaluating patients with ischemic stroke (19).

Although current literature has not been able to clarify the role of echocardiography in the routine examination of patients with ischemic stroke, it is an important investigation in them. It is an easily available, non-invasive, relatively inexpensive method, which is easy to perform in centers that have integrated stroke and cardiology units, providing information that can change both the treatment and the understanding of the etiological mechanism of stroke.

Therefore, the objectives of this study were to assess the following: (1a) Whether or not the percentage of patients with ischemic stroke classified as the undetermined TOAST type decreased as a result of echocardiographic examination, (1b) Whether or not the prognosis after ischemic stroke is worse in patients with an undetermined TOAST classification, and (2) The predictive capacity of echocardiography in determining the prognosis of patients with ischemic stroke.

It was hypothesized that transthoracic echocardiography, in the routine investigation of patients with ischemic stroke, permits better etiological assessment, and consequently, improves the prognosis after the event.

## Materials and Methods

### Study Design

This retrospective cohort study was performed at the Stroke Unit (SU) of the Clinical Hospital of the School of Medicine of Botucatu (HC-FMB-UNESP), and included 1,100 inpatients diagnosed with ischemic stroke. Data collection was conducted at two time points: at hospital admission and 90 days after hospital discharge.

The study was approved by the Research Ethics Committee (REC) of the School of Medicine of Botucatu under no. 2,698,569.

### Study Population

The sample size was estimated based on simple random sampling, with a normal distribution for the numerical outcomes, type I error = 0.05, and, of all possible associations, the association between left ventricular remodeling (one of the echocardiographic examination variables) and an unfavorable modified Rankin scale (mRS) at 90 days to estimate the test power. Based on the descriptive findings obtained from this association, the test power was estimated to be above 80% for the analyzed association, indicating that the sample size to analyze objective 1a (*n* = 1,100), objective 1b (*n* = 994), and objective 2 (*n* = 927) was large enough to ensure test powers >80%.

The study included adults diagnosed with ischemic stroke after clinical evaluation and imaging, such as computed tomography (CT) at admission, and control evaluations, between October 2012 and February 2018.

### Clinical Evaluation

The following data were collected from the electronic medical records of clinical evaluations performed by the assistant medical team during the hospitalization period: age, sex, race (white/non-white), the presence of comorbidities (systemic arterial hypertension, type 2 diabetes mellitus, dyslipidemia, smoking, alcohol use, illicit drug use, arrhythmias such as AF or atrial flutter, and a history of previous stroke), the continuous use of medications [acetylsalicylic acid, clopidogrel, anticoagulants, angiotensin-converting enzyme (ACE) inhibitors, angiotensin II receptor blockers (ARB), and statins].

### Neurological Evaluation

Data pertaining to neurological assessments conducted by the medical team during the hospitalization of the patients at the SU and at 90 days after hospital discharge were collected from the electronic medical records. This data included the score obtained using the National Institutes of Health Stroke Scale (NIHSS) (20) at admission, on hospital discharge, and at 90 days after discharge; the clinical condition classified by the Oxfordshire or Bamford scale (21); the TOAST classification (7); the modified Rankin scale (22) (previous, at hospital discharge, and 90 days after discharge); the recurrence of stroke; the presence of carotid and vertebrobasilar system stenosis and its quantification.

All the variables were obtained from the stroke data bank of Botucatu Medical School. The database is audited monthly by the stroke unit coordinator.

### Echocardiographic Evaluation

The patients underwent TTE during hospitalization at the SU. Transesophageal echocardiography was performed when a right-left intracardiac shunt was suspected on TTE, or in case of other findings that required better diagnostic interpretation.

The following parameters were verified with these examinations: left atrial diameter (LA) (mm), left ventricular mass (LVM) (g), left ventricular ejection fraction by the Teichholz method (LVEF) (%), the presence of alterations in segmental contractility (ASC), the presence of left ventricular hypertrophy (LVH), left ventricle remodeling (LVR), severe diastolic dysfunction (SDD), moderate diastolic dysfunction (MoDD), mild diastolic dysfunction (MiDD), severe aortic valve insufficiency (S AoV Insuf), moderate aortic valve insufficiency (Mo AoV Insuf), mild aortic valve insufficiency (Mi AoV Insuf), severe mitral valve insufficiency (S MiV Insuf), moderate mitral valve insufficiency (Mo MiV Insuf), and mild mitral valve insufficiency (Mi MiV Insuf).

### The Etiological Investigation Protocol in Patients With Ischemic Stroke

The investigation protocol at the institution was based on the TOAST classification. All the patients underwent a brain CT at admission, while some underwent an additional scan after 24 h. Depending on the clinical progression, MRI was done for the patients with posterior circulation events or in those with a doubtful diagnosis.

CT angiography of the cerebral and cervical arteries was performed when the patient arrived within 8 h of the ictus, and duplex ultrasound of the cervical arteries and transcranial Doppler were performed 8 h after the ictus. The study was complemented by an anatomical examination (CT angiography or digital angiography) whenever required.

TTE was conducted to locate a cardioembolic source other than AF, while a transesophageal echocardiogram was requested to assess a right-left circulation shunt, left atrial appendage thrombus, and an atheroma in the thoracic aorta.

All patients underwent electrocardiography at admission followed by 24 h of cardiac monitoring. The 24 h Holter test was performed for patients older than 55 years with suspected arrhythmias, and for cryptogenic strokes.

The patient underwent laboratory investigations for syphilis, Chagas disease, glycated hemoglobin, thyroid stimulating hormone (TSH), total cholesterol and fractions, and triglycerides. An autoimmune panel was also performed for patients aged <55 years.

### Statistical Analysis

Continuous variables were expressed as mean and standard deviation, while categorical variables were presented as absolute values and percentages. The statistical models were built to separately answer each objective defined in the study.

The potential confounders (variables identified in the maximal model that were clinically relevant, with *p* < 0.20) considered for all the objectives of this study were as follows: age; sex; race; systemic arterial hypertension; type 2 diabetes mellitus; dyslipidemia; smoking; alcoholism; the use of illicit drugs; AF; previous stroke; the continuous use of acetylsalicylic acid, clopidogrel, anticoagulant, ACEI, ARB, and statins; NIHSS at admission; TOAST classification at admission; mRS at admission.

The association between the echocardiographic examination and being classified as the undetermined TOAST type was analyzed using the multiple logistic regression model, including the potential pre-established confounders. The variables included were those presenting statistical significance in the univariate analysis (Objective 1a).

To verify the association between the classification as the undetermined TOAST type and the NIHSS scale score at discharge and 90 days after hospital discharge, the multiple linear regression model was used independently after adjusting for potential confounders. The multiple logistic regression model, adjusted for potential pre-established confounders, was also used to verify the association between the classification as the undetermined TOAST type and unfavorable mRS (mRS > 3 at discharge and 90 days after hospital discharge), and in-hospital mortality. The variables included were those presenting statistical significance in the univariate analysis (Objective 1b).

The association between the echocardiographic variables previously described and the NIHSS scale score at discharge and 90 days after hospital discharge was analyzed using the multiple linear regression model independently and adjusted for potential confounders. The multiple logistic regression model was used to verify the association between the echocardiographic variables and unfavorable mRS scores (mRS > 3) at discharge and 90 days after hospital discharge, and in-hospital mortality. The models were adjusted for potential pre-established confounders. The variables included were those presenting statistical significance in the univariate analysis (Objective 2).

A comparison between the TOAST types and the echocardiographic variables was performed using the Kruskal-Wallis non-parametric test, followed by the Dunn test for multiple comparisons. Statistical significance was set at *p* < 0.05. The analysis was performed using the SPSS version 21 software.

## Results

### Patient Inclusion in the Study

**Figure 1:**
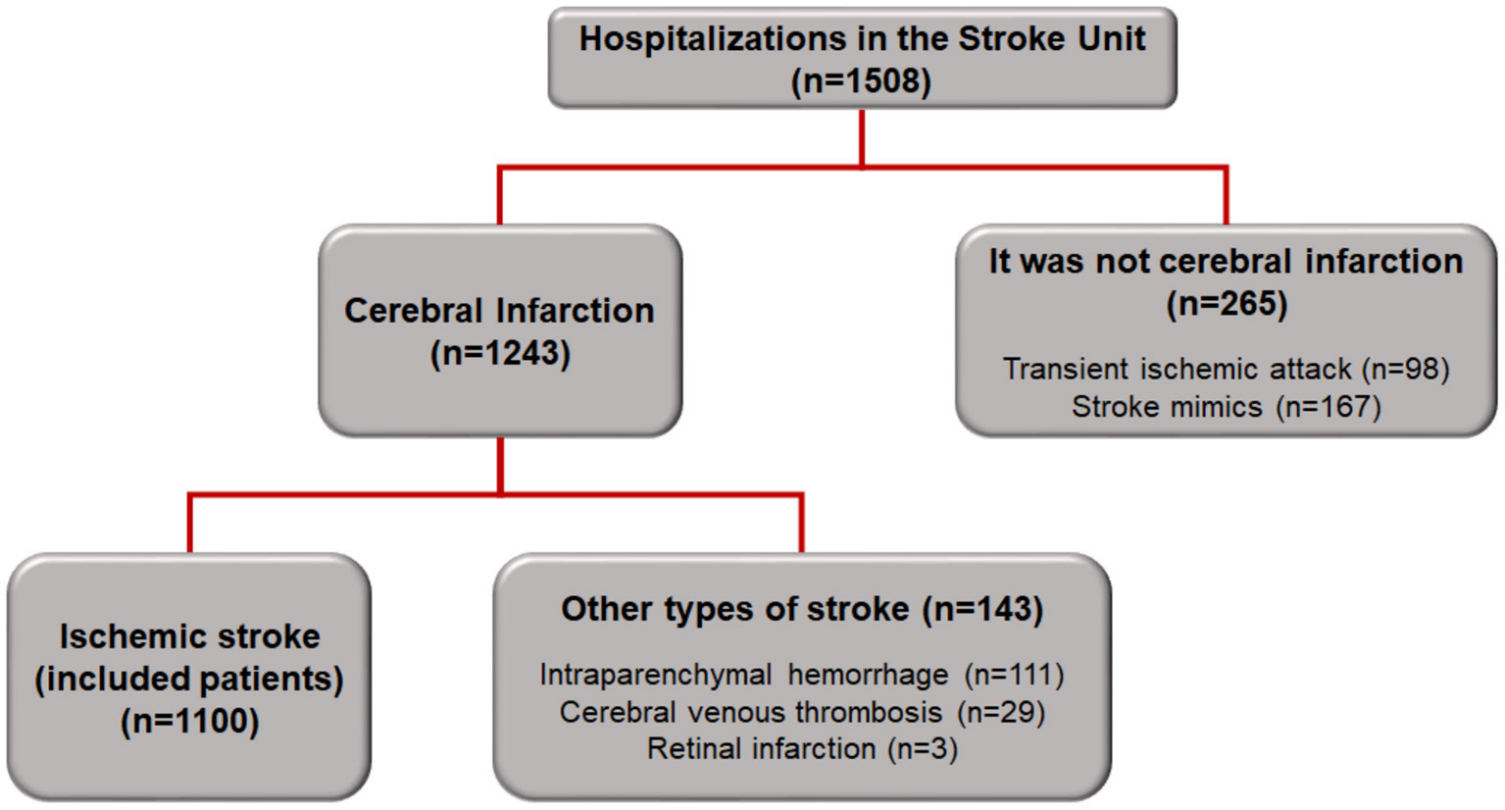
Flowchart of patient inclusion in the study.

A total of 1,508 patients were admitted to the SU between October 2012 and February 2018. Of these, 1,243 patients had a confirmed diagnosis of cerebral infarction, and the 1,100 patients diagnosed with ischemic stroke were included in this study (Figure 1).

### Demographic and Clinical Characteristics of Patients Admitted With Ischemic Stroke

Table 1 shows the demographic characteristics of the patients admitted with ischemic stroke, as well as the neurological assessments regarding the TOAST classification, Bamford clinical classification, and the degree of disability using the modified Rankin scale. Echocardiography was performed in 977 patients (88.82%).

**Table 1:** Demographic and clinical characteristics of patients admitted with ischemic stroke (*n* = 1,100).

**Variables**	***n*** **(%)**
Male	606 (55.09)
Age (years)	68.1 ± 13.3
Race (non-white)	107 (9.73)
History of previous stroke	103 (9.36)
NIHSS at admission	8.37 ± 7.44
Echocardiogram	977 (88.82)
**Neurological evaluation**	
Undetermined TOAST	448 (40.7)
Cardioembolic TOAST	255 (23.2)
Large vessel TOAST	164 (14.9)
Small vessel TOAST	181 (16.5)
TOAST due to other causes	52 (4.7)
LACS ischemic stroke	416 (37.8)
PACS ischemic stroke	346 (31.5)
POCS ischemic stroke	141 (12.8)
TACS ischemic stroke	197 (17.9)
Previous mRS 0	816 (74.2)
Previous mRS 1	159 (14.5)
Previous mRS 2	51 (4.6)
Previous mRS 3	53 (4.8)
Previous mRS 4	19 (1.7)
Previous mRS 5	2 (0.2)

*Values are expressed as numbers and percentages or as mean and standard deviation. NIHSS, National Institute of Health Stroke Scale; TOAST, Trial of Org 10172 in Acute Stroke Treatment; LACS, lacunar syndromes; PACS, partial anterior circulation syndromes; POCS, posterior circulation syndromes; TACS, total anterior circulation syndromes; mRS, Modified Rankin scale*.

Table 2 shows the risk factors for cardiovascular diseases and the medications used being at the time of hospitalization.

**Table 2:** Risk factors for cardiovascular diseases and medications being used previously in patients admitted to the Stroke Unit (*n* = 1,100 patients).

**Variables**	***n*** **(%)**
**Risk factors for CVD**	
Systemic arterial hypertension	838 (76.18)
Diabetes mellitus	383 (34.82)
Dyslipidemia	191 (17.36)
Atrial fibrillation	203 (18.45)
Smoking	479 (43.55)
Alcoholism	256 (23.27)
Use of illicit drugs	11 (1.00)
**Previously used medications**	
Acetylsalicylic acid	302 (27.45)
Clopidogrel	45 (4.09)
Oral anticoagulant	54 (4.91)
ACEI or ARB	470 (42.73)
Statins	300 (27.27)

*Values are expressed as numbers and percentages. CVD, cardiovascular disease; ACEI, angiotensin-converting enzyme inhibitor; ARB, angiotensin II receptor blocker*.

### Association Between Echocardiography and Classification as the Undetermined TOAST Type (Objective 1a)

Table 3 shows that patients undergoing TTE were 3.1 times less likely to be classified as the undetermined TOAST type (OR = 0.32; 95% CI: 0.21-0.51; *p* < 0.001).

**Table 3:** Multiple logistic regression model adjusted to explain the chance of classification as the undetermined TOAST type on echocardiography, corrected for confounding variables (*n* = 1,100 patients).

**Variables**	**OR**	**95% CI**		***p***
Age (years)	1.014	1.003	1.025	0.010
Sex (male)	0.781	0.592	1.031	0.081
Race (non-white)	1.482	0.955	2.301	0.079
Dyslipidemia	0.689	0.483	0.985	0.041
Smoking	0.848	0.641	1.121	0.247
Atrial fibrillation	0.087	0.054	0.143	0.000
History of previous stroke	1.424	0.897	2.260	0.133
LACS ischemic stroke				0.000
PACS ischemic stroke	2.360	1.706	3.266	0.000
POCS ischemic stroke	1.928	1.255	2.964	0.003
TACS ischemic stroke	2.161	1.450	3.220	0.000
Previous mRS	1.197	1.028	1.395	0.021
**Echocardiogram**	**0.324**	**0.206**	**0.510**	**<0.001**

*A parsimonious model, including only variables with p < 0.20, in the multiple logistic regression for the chance of being classified as the undetermined TOAST type that showed an association. TOAST, Trial of Org 10172 in Acute Stroke Treatment; OR, odds ratio; 95% CI, 95% confidence interval; LACS, lacunar syndromes; PACS, partial anterior circulation syndromes; POCS, posterior circulation syndromes; TACS, total anterior circulation syndromes; mRS, Modified Rankin scale*.

The number needed to treat was calculated to be 3.4, implying that for every 3.4 TTEs performed, one patient would be prevented from being classified as the undetermined TOAST type.

### Association Between Being Classified as the Undetermined TOAST Type and the Outcomes at Discharge and at 90 Days After Hospital Discharge (Objective 1b)

There was no association between being classified as the undetermined TOAST type and the outcomes at hospital discharge, as can be seen from the following: NIHSS score (β: −0.040; *p* = 0.871) and mRS score >3 (OR: 0.901; *p* = 0.544) using the multiple linear regression model corrected for confounding variables (alcoholism, history of previous stroke, TACS ischemic stroke, POCS ischemic stroke, PACS ischemic stroke, echocardiogram, age, and NIHSS at admission), and by the multiple logistic regression model corrected for confounding variables (age, male, non-white race, diabetes mellitus, alcoholism, use of oral anticoagulants, NIHSS at admission, LACS ischemic stroke, PACS ischemic stroke, POCS ischemic stroke, and TACS ischemic stroke).

With regard to the prognosis 90 days after hospital discharge, no association was found between the classification as the undetermined TOAST type and NIHSS score outcomes (β: −0.160; *p* = 0.560) and mRS score >3 (OR: 0.812; *p* = 0.261) 90 days after hospital discharge, using the multiple linear regression model corrected for confounding variables (AF, history of previous stroke, use of clopidogrel, use of oral anticoagulants, TACS ischemic stroke, POCS ischemic stroke, PACS ischemic stroke, echocardiogram, age, NIHSS at admission, previous mRS), and the multiple logistic regression model corrected for confounding variables (age, male sex, diabetes mellitus, dyslipidemia, alcoholism, use of oral anticoagulants, NIHSS at admission, LACS ischemic stroke, PACS ischemic stroke, POCS ischemic stroke, TACS ischemic stroke, previous mRS, and echocardiogram).

Undergoing an echocardiogram was a protective factor against death during hospitalization, and reduced the possibility of in-hospital death by 11.1 times (OR: 0.090; *p* < 0.001). Conversely, being classified as the undetermined TOAST type increased the chances of mortality during hospitalization by 2.0 times (OR: 2.00; *p* = 0.013), as shown in Table 4.

**Table 4:** Multiple logistic regression model to explain in-hospital mortality due to the undetermined TOAST type classification, corrected for confounding variables (*n* = 1,100 patients).

**Variables**	**OR**	**95% CI**	***p***
Age (years)	1.022	1.000–1.050	0.033
Male	1.870	1.070–3.260	0.028
SAH	1.880	0.930–3.800	0.078
Diabetes	1.540	0.860–2.750	0.143
Use of ASA	1.450	0.820–2.580	0.205
Use of oral anticoagulant	0.250	0.050–1.290	0.099
NIHSS at admission	1.110	1.070–1.150	<0.001
Bamford			<0.001
PACS ischemic stroke	14.560	1.860–114.110	0.011
TACS ischemic stroke	28.550	3.480–234.550	0.002
LACS ischemic stroke	40.530	5.050–325.310	<0.001
**Echocardiogram**	**0.090**	**0.050–0.170**	**<0.001**
**Undetermined TOAST**	**2.000**	**1.160–3.460**	**0.013**

*TOAST, Trial of Org 10172 in Acute Stroke Treatment; OR, odds ratio; 95% CI, 95% confidence interval; SAH, systemic arterial hypertension; ASA, acetylsalicylic acid; NIHSS, National Institutes of Health Stroke Scale; PACS, partial anterior circulation syndrome; TACS, total anterior circulation syndrome; LACS, lacunar syndrome; POCS, posterior circulation syndrome*.

### Association Between Echocardiographic Variables and Outcomes at Discharge and at 90 Days After Hospital Discharge (Objective 2)

There was no association between the echocardiographic variables and NIHSS score outcomes (Table 5) and mRS > 3 (Table 6) at discharge.

**Table 5:** Association between the echocardiographic variables of patients hospitalized due to ischemic stroke and NIHSS scores at hospital discharge (*n* = 977 patients).

**Variables**	**β**	**95% CI**	***p***
(Intercept)	−2.125	−5.019–0.770	0.150
LA (mm)	0.004	−0.042–0.050	0.861
LVM (g)	−0.002	−0.007–0.004	0.554
LVEF (%)	0.012	−0.012–0.037	0.328
ASC	0.108	−0.853–1.069	0.826
LVH	0.635	−0.031–1.301	0.062
LVR	0.158	−0.507–0.824	0.641
SDD	−1.238	−3.841–1.366	0.351
MoDD	−0.506	−1.741–0.728	0.421
MiDD	0.077	−0.455–0.610	0.776
S AoV Insuf	−0.179	−2.472–2.113	0.878
Mo AoV Insuf	−0.366	−1.773–1.041	0.610
Mi AoV Insuf	−0.051	−0.673–0.571	0.873
Thrombus in the LA	−1.703	−5.330–1.924	0.357

*The parsimonious model included only variables with p < 0.20, in the multiple linear regression for the NIHSS score at discharge that showed an association. NIHSS, National Institutes of Health Stroke Scale; 95% CI, 95% confidence interval; LA, left atrium diameter; LVM, left ventricular mass; LVEF, left ventricular ejection fraction by the Teichholz method; ASC, alteration of segmental contractility; LVH, left ventricular hypertrophy; LVR, left ventricle remodeling; SDD, severe diastolic dysfunction; MoDD, moderate diastolic dysfunction; MiDD, mild diastolic dysfunction; S AoV Insuf, severe aortic valve insufficiency; Mo AoV Insuf, moderate aortic valve insufficiency; Mi AoV Insuf, mild aortic valve insufficiency*.

*Model adjusted for potential confounders: alcoholism, the use of anticoagulants, Bamford classification, age, and the NIHSS and mRS scores at admission*.

**Table 6:** Association between the echocardiographic variables of patients hospitalized due to ischemic stroke and mRS > 3 at hospital discharge (*n* = 977 patients).

**Variables**	**OR**	**95% CI**	***p***
LA (mm)	0.992	0.958–1.028	0.663
LVM (g)	1.001	0.997–1.005	0.600
LVEF (%)	1.000	0.982–1.018	0.986
ASC	1.386	0.678–2.834	0.371
LVR	1.516	0.907–2.532	0.112
LVH	1.344	0.803–2.248	0.260
SDD	1.172	0.779–1.765	0.446
MoDD	1.209	0.463–3.157	0.699
MiDD	0.473	0.053–4.209	0.502
S AoV Insuf	1.119	0.700–1.787	0.639
Mo AoV Insuf	0.846	0.285–2.509	0.763
Mi AoV Insuf	0.481	0.090–2.578	0.393
Thrombus in the LA	0.520	0.035–7.710	0.634

*A parsimonious model, including only variables with p < 0.20, in the multiple logistic regression for the chance of mRS > 3 at discharge showing an association. mRS, Modified Rankin scale; OR, odds ratio; 95% CI, 95% confidence interval; LA, left atrium diameter; LVM, left ventricular mass; LVEF, left ventricular ejection fraction by the Teichholz method; ASC, alteration of segmental contractility; LVH, left ventricular hypertrophy; LVR, left ventricle remodeling; SDD, severe diastolic dysfunction; MoDD, moderate diastolic dysfunction; MiDD, mild diastolic dysfunction; S AoV Insuf, severe aortic valve insufficiency; Mo AoV Insuf, moderate aortic valve insufficiency; Mi AoV Insuf, mild aortic valve insufficiency*.

*Model adjusted for potential confounders: age, sex, alcoholism, previous stroke, use of anticoagulant, NIHSS and mRS at admission, and Bamford classification (PACS, POCS, TACS)*.

At 90 days after hospital discharge, there was no association between the echocardiographic findings and NIHSS score outcomes. Furthermore, there was no association between the echocardiographic findings and mRS>3 score outcomes, except for left ventricular remodeling (OR = 1.78; 95% CI: 1.06–2.98; *p* = 0.028).

An evaluation of the echocardiographic variables and their correlation with the TOAST classification type showed significantly greater values for left atrial diameter and LVM and significantly lower left ventricular ejection fraction values in patients with stroke classified as the cardioembolic TOAST type, as shown in Table 7.

**Table 7:** Comparison between TOAST types and echocardiographic variables (*n* = 977 patients).

	**Other causes**	**Undetermined classification**	**TOAST** **Large vessels**	**Small vessels**	**Cardioembolic**	***p*** ^**(1)**^
LA	37.0 (30.0–52.0)	40.0 (27.0–63.0)	39.0 (29.0–55.0)	39.0 (28.0–58.0)	46.0 (30.0–77.0)	**<0.001**
LVM	141.5 (85.4–364.8)	167.6 (88.8–635.8)	169.3 (85.2–345.5)	173.0 (96.8–401.1)	199.6 (89.7–470.7)	**<0.001**
EF	69.5 (26.0–78.0)	70.0 (24.0–86.0)	70.0 (28.0–86.0)	70.0 (31.0–89.5)	62.0 (20.0–88.0)	**<0.001**

*Values expressed as median (minimum-maximum). (1) Kruskal-Wallis for independent samples. Statistically significant contrasts (p < 0.05; Dunn's test for independent samples): LA, Other causes, Undetermined, Large vessels, Small vessels < Cardioembolic; LVM, Other causes, Undetermined, Large vessels, Small vessels < Cardioembolic, Other causes < Small, Large vessels; EF, Other causes, Undetermined, Large vessels, Small vessels < Cardioembolic. TOAST, Trial of Org 10172 in Acute Stroke Treatment; Med, median; Min, minimum value; Max, maximum value; LA, left atrium diameter (mm); LVM, left ventricular mass (g); LVEF, left ventricular ejection fraction by the Teichholz method (%)*.

## Discussion

In the present study, echocardiography during hospitalization due to ischemic stroke reduced the possibility of being classified as the undetermined TOAST type and was associated with lower in-hospital mortality.

The distribution of the different TOAST classifications was similar to that reported previously in literature; >30% (40.7%) of the patients were classified as the undetermined type, despite the investigation protocol (23). The incidence of small vessel TOAST classification was 16.45%, that of large vessels was 14.81%, and that of other causes was 4.72%. Of the 23.14% patients classified as the cardioembolic type, the main associated factor was the presence of AF (in 18% of all ischemic strokes), with a higher incidence than seen in previous studies on Brazilian patients (24).

In this study, echocardiography decreased the number of patients classified without a defined etiology, and this relationship confirmed the study hypothesis. TTE is a non-invasive and low-cost examination, and the association described above proves the importance of including it in an investigation protocol for patients hospitalized with ischemic stroke.

Although echocardiography did not correlate with a better patient prognosis, as measured by the NIHSS and Rankin scale scores, both at discharge and after 90 days, it increased the chance of identifying specific TOAST classifications, thereby decreasing the chance of inappropriate patient treatment. Although the highest NIHSS was found among patients who did not undergo echocardiography, this variable was taken into account and adjusted in the multiple regression for the mortality outcome. The correlation between echocardiography and the lower frequency of death at admission reinforces the importance of this examination for proper patient management.

The 5 year survival probability was higher in patients with small artery occlusion stroke (73.8 [95% CI, 70.4–77.3]) and lower in patients with cardioembolic stroke (40.9 [95% CI, 37.2–45.0]) and in indeterminate stroke patients (50.3 [95% CI, 47.2–53.7]) (10).

There was no association between the variables assessed on the echocardiogram and the NIHSS and mRS scores at 90 days. These data do not corroborate with those of studies that evaluated systolic function through ejection fraction and diastolic dysfunction and reported a worse outcome in stroke (25, 26). Ventricular mass was reported to be a risk factor for non-fatal ischemic stroke (27), as well as for recurrence and death in cases of severe LVH (28).

A possible reason for this difference in the correlation between the echocardiogram measurements and prognosis in previous literature is the evaluation of these variables without considering the TOAST etiological classification. Patients with a cardioembolic TOAST classification generally present with more changes in the echocardiographic measurements. In this study, for example, patients with a cardioembolic TOAST classification had a higher ventricular mass, larger atrial diameter, and a lower EF. Thus, such echocardiogram findings are possibly collinear with a cardioembolic TOAST classification and are not independent prognostic factors.

A large number of patients with ischemic stroke were on antiplatelet therapy (31.54%). A comprehensive investigation of these patients can help in identifying conditions in which anticoagulation would be the appropriate prophylaxis; echocardiography may have an important role here. Thus, a thorough investigation of the patient is important for the proper characterization and management of ischemic stroke. Emerging evidence suggests that atrial enlargement may be a biomarker of AF-independent underlying thrombogenic atrial heart disease with an independent risk of indeterminate or recurrent cardioembolic stroke (17).

Despite its findings, this study has its limitations. It is a retrospective study involving a single center only, making it impossible to obtain the measurement of the left atrial volume for analysis. Left atrial volume has been shown to be a powerful prognostic variable in heart disease.

We also understand as a limitation that the design of this study did not allow the identification of mechanisms by which the echocardiogram correlated with a reduction in mortality, which opens up frontiers for future studies in this area.

Based on the results of the present study, we can conclude that echocardiography during hospitalization for ischemic stroke may be associated with a decreased chance of an undetermined TOAST classification, and also with lower mortality during hospitalization. On the other hand, an undetermined TOAST classification may correlate with higher mortality during hospitalization, suggesting the importance of including echocardiography in the hospital investigation protocol for patients with ischemic stroke.

## Data Availability Statement

The original contributions presented in the study are included in the article/Supplementary Material, further inquiries can be directed to the corresponding author/s.

## Ethics Statement

The studies involving human participants were reviewed and approved by Research Ethics Committee (REC) of the School of Medicine of Botucatu under no. 2,698,569. The patients/participants provided their written informed consent to participate in this study.

## Author Contributions

RT contributed to the literature search, study design, data collection, data analysis and interpretation, and writing of the manuscript. GS, GM, and ST participated in the literature search, study design, data analysis and interpretation, and in the writing of the manuscript. JS, GL, LM, and RB conducted the literature search, data analysis and interpretation, and wrote the manuscript. HN and SZ participated in the literature search, study design, data analysis and interpretation, and wrote the manuscript. All authors contributed to manuscript revision, read, and approved the submitted version.

## Conflict of Interest

The authors declare that the research was conducted in the absence of any commercial or financial relationships that could be construed as a potential conflict of interest.

## Publisher's Note

All claims expressed in this article are solely those of the authors and do not necessarily represent those of their affiliated organizations, or those of the publisher, the editors and the reviewers. Any product that may be evaluated in this article, or claim that may be made by its manufacturer, is not guaranteed or endorsed by the publisher.

## Abbreviations

TOAST: Trial of Org 10172 in Acute Stroke Treatment
TTE: transthoracic echocardiography
mRS: Modified Rankin scale
OR: odds ratio
95% CI: 95% confidence interval
LA: left atrium diameter
LVM: left ventricular mass
LVEF: left ventricular ejection fraction by the Teichholz method
ASC: alteration of segmental contractility
LVH: left ventricular hypertrophy
LVR: left ventricle remodeling
SDD: severe diastolic dysfunction
MoDD: moderate diastolic dysfunction
MiDD: mild diastolic dysfunction
S AoV Insuf: severe aortic valve insufficiency
Mo AoV Insuf: moderate aortic valve insufficiency
Mi AoV Insuf: mild aortic valve insufficiency
NIHSS: National Institute of Health Stroke Scale
LACS: lacunar syndromes
PACS: partial anterior circulation syndromes
POCS: posterior circulation syndromes
TACS: total anterior circulation syndromes
ACEI: angiotensin-converting enzyme inhibitor
ARB: angiotensin II receptor blocker.
