# Supplementary material for: The Role of Transthoracic Echocardiography in the Evaluation of Patients With Ischemic Stroke
Source: Front Cardiovasc Med. 2021 Aug 27;8:710334. doi: 10.3389/fcvm.2021.710334 (PMC8432611; doi:10.3389/fcvm.2021.710334)
Supplement: Supplementary file 1 [file Data_Sheet_1.pdf]

SUPPLEMENTARY MATERIAL

The univariate analyses that preceded the multiple regression models in Tables 3, 4, 5 and 6 are presented below.

Univariate analysis for the model in Table 3

Variables	OR	95% CI		*p*
Age (years)	1.02	1.00	1.03	0.007
Sex (male)	0.72	0.54	0.97	0.032
Race (non-white)	1.53	0.98	2.39	0.059
Systemic arterial hypertension	0.98	0.69	1.38	0.894
Diabetes mellitus	0.82	0.61	1.11	0.206
Dyslipidemia	0.69	0.47	1.01	0.056
Smoking	0.80	0.60	1.08	0.146
Alcoholism	1.20	0.84	1,73	0.321
Use of illicit drugs	0.94	0.24	3.64	0.925
Atrial fibrillation	0.09	0.05	0.15	0.000
History of previous stroke	1.45	0.91	2.32	0.118
Acetylsalicylic acid	1.03	0.70	1.51	0.899
Clopidogrel	0.94	0.46	1.92	0.875
Oral anticoagulant	0.78	0.35	1.77	0.558
ACEI or ARB	0.81	0.58	1.13	0.211
Statins	1.29	0.86	1.95	0.217
NIHSS at admission	0.99	0.97	1.02	0.606
Bamford (Ref: LACS)				0.000
PACS ischemic stroke	2.40	1.68	3.43	0.000
POCS ischemic stroke	1.95	1.26	3.02	0.003
TACS ischemic stroke	2.34	1.37	3.99	0.002
Previous mRS	1.20	1.03	1.41	0.020
Echocardiogram	0.32	0.20	0.50	0.000

OR: odds ratio; 95% CI: 95% confidence interval; ACEI: angiotensin-converting enzyme inhibitor; ARB: angiotensin II receptor blocker; NIHSS: National Institutes of Health Stroke Scale; PACS: partial anterior circulation syndromes; POCS: posterior circulation syndromes; TACS: total anterior circulation syndromes; mRS: Modified Rankin scale.

Univariate analysis for the model in Table 4

Variables	OR	95% CI		*p*
Age (years)	1.02	1.00	1.05	0.077
Male	2.19	1.19	4.03	0.012
Race (non-white)	1.01	0.43	2.40	0.974
Systemic arterial hypertension	2.02	0.93	4.36	0.075
Diabetes	1.56	0.86	2.82	0.144
Dyslipidemia	1.22	0.59	2.56	0.590
Smoking	0.86	0.45	1.65	0.656
Alcoholism	0.78	0.35	1.74	0.541
Use of illicit drugs	0.00	0.00		0.999
Atrial fibrillation	1.44	0.68	3.06	0.345
History of previous stroke	1.68	0.66	4.31	0.277
Acetylsalicylic acid	1.78	0.88	3.63	0.110
Clopidogrel	0.38	0.06	2.42	0.307
Oral anticoagulant	0.21	0.03	1.28	0.090
ACEI or ARB	0.80	0.41	1.53	0.494
Statins	0.82	0.38	1.77	0.616
NIHSS at admission	1.11	1.07	1.16	0.000
Bamford				0.001
PACS ischemic stroke	14.05	1.79	110.03	0.012
TACS ischemic stroke	26.48	3.23	217.35	0.002
LACS ischemic stroke	36.40	4.50	294.63	0.001
mRS at admission	0.86	0.66	1.12	0.258
Echocardiogram	0.08	0.04	0.16	0.000
Undetermined TOAST	2.38	1.24	4.55	0.009

OR: odds ratio; 95% CI: 95% confidence interval; ACEI/ARB: angiotensin-converting enzyme inhibitor/angiotensin II receptor blocker; NIHSS: National Institutes of Health Stroke Scale; PACS: partial anterior circulation syndromes; TACS: total anterior circulation syndromes; LACS: lacunar syndromes; mRS: Modified Rankin scale; TOAST: Trial of Org 10172 in Acute Stroke Treatment.

Univariate analysis for the model in Table 5

Variables	β	95% CI		*p*
Male	-0.293	-0.844	0.258	0.297
Race (non-white)	0.265	-0.550	1.080	0.524
Age (years)	0.024	0.003	0.044	0.025
NIHSS at admission	0.567	0.519	0.615	0.000
mRS at admission	0.429	0.147	0.710	0.003
Systemic arterial hypertension	-0.196	-0.826	0.433	0.541
Diabetes	0.046	-0.484	0.576	0.865
Dyslipidemia	-0.315	-0.968	0.338	0.345
Smoking	0.113	-0.402	0.629	0.666
Alcoholism	0.543	-0.074	1.161	0.084
Use of illicit drugs	0.540	-1.757	2.838	0.645
Atrial fibrillation	-0.396	-1.115	0.322	0.280
History of previous stroke	0.348	-0.460	1.157	0.399
Acetylsalicylic acid	0.287	-0.397	0.971	0.411
Clopidogrel	0.528	-0.656	1.713	0.382
Oral anticoagulant	-0.910	-2.106	0.285	0.136
ACEI or ARB	-0.075	-0.645	0.496	0.797
Statins	0.202	-0.519	0.923	0.583
POCS ischemic stroke	2.462	1.470	3.454	0.000
LACS ischemic stroke	0.164	-0.602	0.930	0.674
TACS ischemic stroke	0.564	-0.050	1.178	0.072
PACS ischemic stroke	0[a]			
**Echocardiogram variables**				
LA (mm)	0.031	-0.019	0.080	0.229
LVM (g)	-0.002	-0.007	0.004	0.610
LVEF (%)	0.003	-0.022	0.028	0.787
ASC	0.053	-0.917	1.023	0.915
Mo LVH	0.672	-0.013	1.358	0.055
Mi LVH	0.189	-0.480	0.858	0.580
LVH	0[a]			
SDD	-0.644	-3.291	2.003	0.634
MoDD	-0.567	-1.809	0.675	0.371
MiDD	0.018	-0.522	0.558	0.948
DD	0[a]			
S MiV Insuf	-0.647	-3.662	2.368	0.674
Mo MiV Insuf	-1.267	-2.451	-0.083	0.036
Mi MiV Insuf	-0.900	-1.580	-0.220	0.009
MiV	0[a]			
S AoV Insuf	-0.252	-2.577	2.072	0.832
Mo AoV Insuf	-0.072	-1.490	1.347	0.921
Mi AoV Insuf	0.157	-0.481	0.795	0.630
AoV	0[a]			
Thrombus in the LA	-1.760	-5.400	1.881	0.343

NIHSS: National Institutes of Health Stroke Scale; mRS: Modified Rankin Scale; ACEI/ARB: angiotensin-converting enzyme inhibitor/angiotensin II receptor blocker; POCS: posterior circulation syndrome; LACS: lacunar syndrome; TACS: total anterior circulation syndrome; PACS: partial anterior circulation syndrome; LA: left atrium diameter; LVM: left ventricular mass; LVEF: left ventricular ejection fraction by the

Teichholz method; ASC: alteration of segmental contractility; LVH: left ventricular hypertrophy; SDD: severe diastolic dysfunction; MoDD: moderate diastolic dysfunction; MiDD: mild diastolic dysfunction; S MiV Insuf: severe mitral valve insufficiency; Mo MiV Insuf: moderate mitral valve insufficiency; Mi MiV Insuf: mild mitral valve insufficiency; S AoV Insuf: severe aortic valve insufficiency; Mo AoV Insuf: moderate aortic valve insufficiency; Mi AoV Insuf: mild aortic valve insufficiency.

Univariate analysis for the model in Table 6

Variables	OR	95% CI		*p*
Age (years)	1.020	1.003	1.036	0.018
Male	0.624	0.408	0.952	0.029
Alcoholism	1.706	1.097	2.655	0.018
History of previous stroke	1.501	0.845	2.667	0.166
Oral anticoagulant	0.507	0.196	1.311	0.161
NIHSS at admission	1.263	1.210	1.318	0.000
Bamford				0.220
PACS ischemic stroke	1.156	0.730	1.832	0.537
TACS ischemic stroke	1.630	0.910	2.920	0.100
LACS ischemic stroke	1.759	0.864	3.584	0.120
mRS at admission	2.357	1.862	2.983	0.000
**Echocardiogram variables**				
LA (mm)	0.992	0.958	1.028	0.663
LVM (g)	1.001	0.997	1.005	0.600
LVEF (%)	1.000	0.982	1.018	0.986
ASC	1.386	0.678	2.834	0.371
LVH				0.250
Mi LVH	1.516	0.907	2.532	0.112
Mo LVH	1.344	0.803	2.248	0.260
DD				0.753
MiDD	1.172	0.779	1.765	0.446
MoDD	1.209	0.463	3.157	0.699
SDD	0.473	0.053	4.209	0.502
AoV				0.769
Mi AoV Insuf	1.119	0.700	1.787	0.639
Mo AoV Insuf	0.846	0.285	2.509	0.763
S AoV Insuf	0.481	0.090	2.578	0.393
Thrombus in the LA	0.520	0.035	7.710	0.634

NIHSS: National Institute of Health Stroke Scale; mRS: Modified Rankin Scale; PACS: partial anterior circulation syndrome; TACS: total anterior circulation syndrome; LACS: lacunar syndrome; LA: left atrial diameter; LVM: left ventricular mass; LVEF: left ventricular ejection fraction by the Teichholz method; ASC: alteration of segmental contractility; LVH: left ventricular hypertrophy; MiDD: mild diastolic dysfunction; MoDD: moderate diastolic dysfunction; SDD: severe diastolic dysfunction; Mi AoV Insuf: mild aortic valve insufficiency; Mo AoV Insuf: moderate aortic valve insufficiency; S AoV Insuf: severe aortic valve insufficiency.

COMPARISON BETWEEN THOSE WHO UNDERWENT ECHOCARDIOGRAPHY AND THOSE WHO DID NOT

	Echocardiogram performance				
**Variables**	**No (n=123)**		**Yes (n=977)**		***p***
Age (years)	75 (22-97)		69 (21-102)		0.002
NIHSS at admission	14 (0-37)		5 (0-35)		< 0.001
Previous mRS	0 (0-4)		0 (0-5)		0.044
Male (%)	57	46.3	549	56.2	0.043
Race (non-white) (%)	15	12.2	92	9.4	0.332
Systemic arterial hypetension (%)	83	67.5	755	77.3	0.019
Diabetes mellitus (%)	42	34.1	341	34.9	0.920
Dyslipidemia (%)	21	17.1	170	17.4	1.000
Smoking (%)	37	30.1	442	45.2	0.001
Alcoholism (%)	21	17.1	235	24.1	0.090
Use of ilicit drugs (%)	1	0.8	10	1.0	1,000
Atrial fibrillation (%)	22	17.9	181	18.5	0.903
History of previous stroke (%)	6	4.9	97	9.9	0.072
Acetylsalicylic acid (%)	27	22.0	275	28.1	0.164
Clopidogrel (%)	3	2.4	42	4.3	0.468
Oral anticoagulant (%)	10	8.1	44	4.5	0.116
ACEI or ARB (%)	50	40.7	420	43.0	0.630
Statins (%)	32	26.0	268	27.4	0.749
Undetermined TOAST (%)	82	66.7	366	37.5	<0.001

Values expressed as medians and interquartile ranges (25-75%) and as number and percentage. NIHSS: National Institute of Health Stroke Scale; mRS: Modified Rankin scale; ACEI: angiotensin-converting enzyme inhibitor; ARB: angiotensin II receptor blocker; TOAST: Trial of Org 10172 in Acute Stroke Treatment. Mann-Whitney test for numerical variables and Chi-square or Fisher’s exact tests for categorical variables.
